# The transcription factor NF-YA is crucial for neural progenitor maintenance during brain development

**DOI:** 10.1016/j.jbc.2024.105629

**Published:** 2024-01-08

**Authors:** Tomoyuki Yamanaka, Masaru Kurosawa, Aya Yoshida, Tomomi Shimogori, Akiko Hiyama, Sankar N. Maity, Nobutaka Hattori, Hideaki Matsui, Nobuyuki Nukina

**Affiliations:** 1 Department of Neuroscience of Disease, Brain Research Institute, Niigata University, Niigata, Japan; 2 Laboratory of Structural Neuropathology, Doshisha University Graduate School of Brain Science, Kyoto, Japan; 3 Laboratory for Molecular Mechanisms of Brain Development, RIKEN Center for Brain Science, Saitama, Japan; 4 Department of Neuroscience for Neurodegenerative Disorders, Juntendo University Graduate School of Medicine, Tokyo, Japan; 5 Department of Genitourinary Medical Oncology, The University of Texas MD Anderson Cancer Center, Houston, Texas, USA; 6 Department of Neurology, Juntendo University Graduate School of Medicine, Tokyo, Japan

**Keywords:** neurogenesis, neural progenitors, transcription factor, alternative splicing, cell death

## Abstract

In contrast to stage-specific transcription factors, the role of ubiquitous transcription factors in neuronal development remains a matter of scrutiny. Here, we demonstrated that a ubiquitous factor NF-Y is essential for neural progenitor maintenance during brain morphogenesis. Deletion of the NF-YA subunit in neural progenitors by using nestin-cre transgene in mice resulted in significant abnormalities in brain morphology, including a thinner cerebral cortex and loss of striatum during embryogenesis. Detailed analyses revealed a progressive decline in multiple neural progenitors in the cerebral cortex and ganglionic eminences, accompanied by induced apoptotic cell death and reduced cell proliferation. In neural progenitors, the NF-YA short isoform lacking exon 3 is dominant and co-expressed with cell cycle genes. ChIP-seq analysis from the cortex during early corticogenesis revealed preferential binding of NF-Y to the cell cycle genes, some of which were confirmed to be downregulated following NF-YA deletion. Notably, the NF-YA short isoform disappears and is replaced by its long isoform during neuronal differentiation. Forced expression of the NF-YA long isoform in neural progenitors resulted in a significant decline in neuronal count, possibly due to the suppression of cell proliferation. Collectively, we elucidated a critical role of the NF-YA short isoform in maintaining neural progenitors, possibly by regulating cell proliferation and apoptosis. Moreover, we identified an isoform switch in NF-YA within the neuronal lineage *in vivo*, which may explain the stage-specific role of NF-Y during neuronal development.

In mammals, most of the neurons in the cerebrum are produced during embryogenesis. Neural progenitors generate these neurons, initially through symmetric cell divisions for self-renewal and later through asymmetric cell divisions for neuronal production. Subsequently, the newly generated neurons migrate to appropriate brain regions. Regulated gene expression plays a critical role in the proper development and maintenance of neurons, and developmental stage-specific transcription factors, known as master factors, are essential for these orchestrated expressions (1, 2, 3, 4). However, the involvement of ubiquitous transcription factors in this process has not been fully elucidated due to their continuous expression throughout the neuronal development.

Nuclear transcription factor-Y (NF-Y) is a ubiquitous factor composed of three subunits, NF-YA, NF-YB, and NF-YC. It binds to a general sequence motif, CCAAT, and is known to regulate the expression of diverse genes (5, 6). We have previously demonstrated that the NF-YA protein is sequestered by the mutant protein responsible for Huntington's neurodegenerative disease (7). Furthermore, NF-YA knockout in differentiated neurons resulted in progressive neurodegeneration, accompanied by abnormal protein deposition in the endoplasmic reticulum (ER) (8, 9, 10). NF-Y binds to the promoters of various ER-related genes, including ER chaperones, and their expressions were dysregulated by NF-Y inactivation (8). These observations suggest that NF-Y is critical for neuronal maintenance by regulating ER homeostasis in the postmitotic cells after neuronal differentiation.

On the contrary, a well-known function of NF-Y is cell cycle progression in proliferating cells (5, 6). It regulates the expression of key cell cycle regulators, including cyclins and E2Fs. Inactivation of NF-Y resulted in cell proliferation defects in fibroblasts and tumor cells, leading to apoptotic death of these cells (11, 12). Furthermore, NF-Y plays a critical role in the progression and maintenance of various stem/progenitor cells such as hematopoietic stem cells (13, 14), myoblasts (15, 16), and embryonic stem cells (17, 18, 19). Notably, an NF-YA splicing isoform lacking exon 3 is expressed dominantly in the stem cells (17, 18, 20). This highlights the potential importance of the NF-YA short isoform in the progression and maintenance of stem cells. However, because NF-Y is frequently downregulated during differentiation, it is uncertain whether the isoform switch occurs during *in vivo* differentiation lineage to regulate distinct cellular processes.

Here, we generated the mice lacking NF-YA in neural progenitors by nestin-cre transgene. The mice exhibited a progressive decline and apoptotic death in multiple neural progenitors in the cortex and ganglionic eminences, resulting in a thinner cortex and the total loss of striatum. Moreover, a dynamic alteration in NF-YA splicing from the short to long isoform was observed during neuronal differentiation. The NF-YA short isoform was co-expressed with cell cycle genes in the progenitors, and ChIP-seq analysis revealed that they are direct targets of NF-Y. Finally, exogenous expression of the NF-YA long isoform in neural progenitors led to a decrease in the number of neurons, possibly by suppressing progenitor proliferation. Taken together, these findings suggest that the NF-YA short isoform, primarily expressed in neural progenitors, plays a critical role in progenitor maintenance through expression of cell cycle genes. The switching of NF-YA to the long isoform may be a critical step in modulating progenitor proliferation by NF-Y.

## Results

### Conditional deletion of NF-YA by nestin-cre transgene causes abnormal brain development

**Figure 1:**
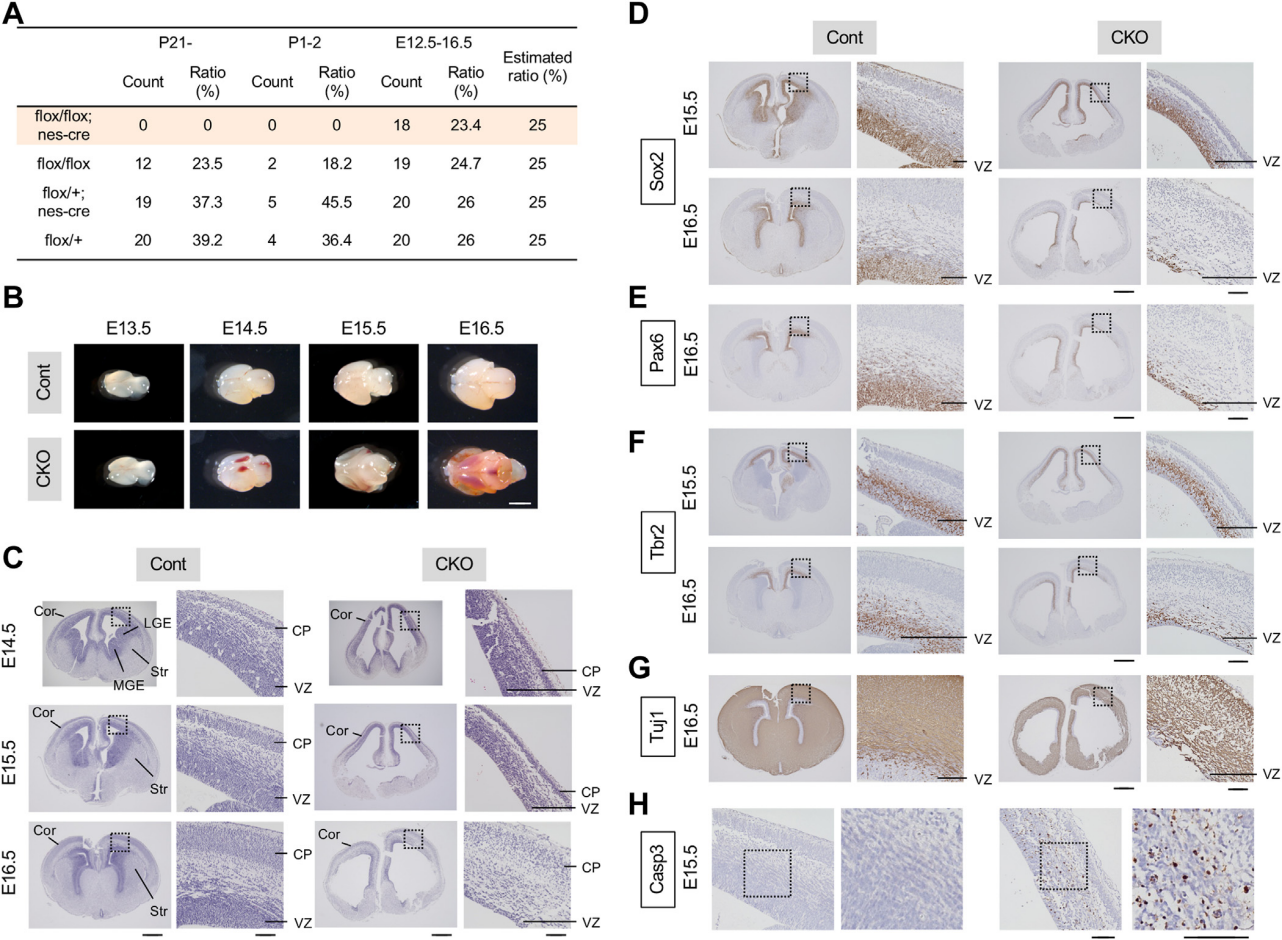
**Abnormal brain morphogenesis and progressive loss of cortical progenitors in NF-YA nes-cko mouse embryos.***A*, ratios of obtained NF-YA nes-cko mice at different stages. NF-YA flox/+; nes-cre mice were crossed with NF-YA flox/flox mice, and obtained mice were sampled at indicated stages and genotyped. NF-YA nes-cko (flox/flox; nes-cre) mice were observed only at embryonic stages at the estimated ratio but unobserved at postnatal stages. *B*, whole brain structure of the NF-YA nes-cko and control mice at different embryonic stages. The brains contained distinct hemorrhages from E14.5 and became translucent in later stages. *C*, HE staining of coronal sections from NF-YA nes-cko and control mice at E14.5 to 16.5. Note the distinct loss of internal brain structures including striata and ganglionic eminences and abnormal development of cortex in nes-cko mice. *D*–*H*, coronal sections from NF-YA nes-cko and control mice at indicated ages were stained with antibodies (*brown*) for Sox2 (a pan-neural apical progenitor marker) (*D*), Pax6 (an apical progenitor marker) (*E*), Tbr2 (an intermediate progenitor marker) (*F*), Tuj1 (a neuronal marker) (*G*), and cleaved caspase-3 (Casp3; an apoptotic cell marker) (*H*), followed by counter-staining with hematoxylin (*blue*). Progressive loss of apical and intermediate progenitors and reduction in Tuj1-positive neurons and a large number of apoptotic cells were observed in the cortex of nes-cko embryos. Magnified images of boxed regions are displayed in adjacent panels. The scale bars represent 2 mm (*B*), 500 μm (C-H for whole brain images), and 100 μm (C-H for magnified images). Cor, cortex; CP, cortical plate; LGE, lateral ganglionic eminence; MGE, medial ganglionic eminence; NF-Y, nuclear transcription factor-Y; Str, striatum; VZ, ventricular zone.

To examine the role of NF-Y in neural development in mice, we generated conditional deletion mice of NF-YA (NF-YA nes-cko) by crossing NF-YA flox mice (12) with nestin-cre transgenic mice that express cre recombinase in neural progenitors (21). Genotyping of the newborn pups revealed no observation of NF-YA nes-cko mice (NF-YA flox/flox; nes-cre) beyond postnatal 21 days (P21) during our routine genotyping (Fig. 1*A*). Additionally, we did not obtain any NF-YA nes-cko pups even at P2 after birth (Fig. 1*A*), indicating that the mice lacking NF-YA are embryonically lethal. We managed to obtain the NF-YA nes-cko embryos at embryonic day 12.5 to 16.5 (E12.5–16.5) at the estimated rate (Fig. 1*A*). Therefore, we concluded that the NF-YA nes-cko mice survive at least up to E16.5 but die before or shortly after birth.

Interestingly, the embryonic brains of NF-YA nes-cko mice exhibited significant structural abnormalities, characterized by translucency and distinct hemorrhages (Fig. 1*B*). Histological analysis of the brains indicated ventricular enlargement and loss of internal brain regions, including the lateral and medial ganglionic eminences (LGE and MGE), along with the developing striatum (Fig. 1*C*). Furthermore, the cortices were thinner and had fewer cells (Fig. 1*C*). No abnormalities were observed in the littermates of other genotypes (NF-YA flox/+; nes-cre, NF-YA flox/flox, or NF-YA flox/+) (Fig. 1, *A*–*C*), which were utilized as control mice in this study. These results indicate that NF-YA deletion in neural progenitors leads to severe defects in brain morphogenesis during embryonic development.

### Conditional deletion of NF-YA results in the progressive loss of cortical progenitors

Because neurogenesis is extensively analyzed in the embryonic cortex and many regulating factors have been so far identified (1, 4), we first focused on this region. In normal development, neural progenitors are reproduced in the ventricular zone (VZ) located at the most apical region. After cell division, neurons are generated from the progenitors and migrate radially toward the cortical plate (CP). In the control mice, the CP was thinner than the VZ containing progenitors at E14.5, but by E16.5, it became thicker due to neurogenesis (Fig. 1*C*). In contrast, although the CP was observed in nes-cko mice at E14.5, it did not enlarge by E16.5, while the VZ was nearly disappeared during these stages (Fig. 1*C*).

To determine the affected cells, we initially stained the tissues with Sox2, an apical progenitor marker in VZ (Fig. S1). In control mice, Sox2 signals were exclusively observed in the VZ. However, these signals became lower at E15.5 and almost diminished at E16.5 in NF-YA nes-cko mice (Fig. 1*D*). Additional data were obtained by staining the tissue with another apical progenitor marker, Pax6 (Fig. 1*E*). Furthermore, Tbr2-positive intermediate progenitors, located in the subventricular zone, manifested a decrease at E16.5 (Fig. 1*F*). These data indicate a progressive loss of neural progenitors in the NF-YA nes-cko cortex during embryogenesis. Subsequently, we stained the tissue with a neuron-specific class III beta-tubulin, Tuj1 (Fig. S1), revealing sparse staining in the cortex of E16.5 NF-YA nes-cko mice compared with the controls (Fig. 1*G*), suggesting that the cortical neurogenesis was compromised in these embryos. Due to the prominence of cells with condensed hematoxylin stain in the NF-YA nes-cko cortex (Fig. 1*C*), we stained the tissue with cleaved caspase-3 (Casp3), a marker of apoptotic cells. As shown in Figure 1*H*, a quantity of Casp3-positive cells was detected in the NF-YA nes-cko cortex at E15.5, while not present in the control cortex, suggesting induction of apoptosis by the NF-YA deletion. The expression of cre was confirmed in the VZ using RNZ reporter mice that express LacZ in nuclei following cre-mediated genome recombination (22) (Fig. S2*A*). Collectively, these data indicate that NF-YA deletion by nestin-cre causes progressive degeneration of neural progenitors accompanied by apoptotic cell death, resulting in abnormal neurogenesis and cortical development in mouse embryos.

### Ganglionic eminence degeneration and striatum loss by NF-YA deletion

**Figure 2:**
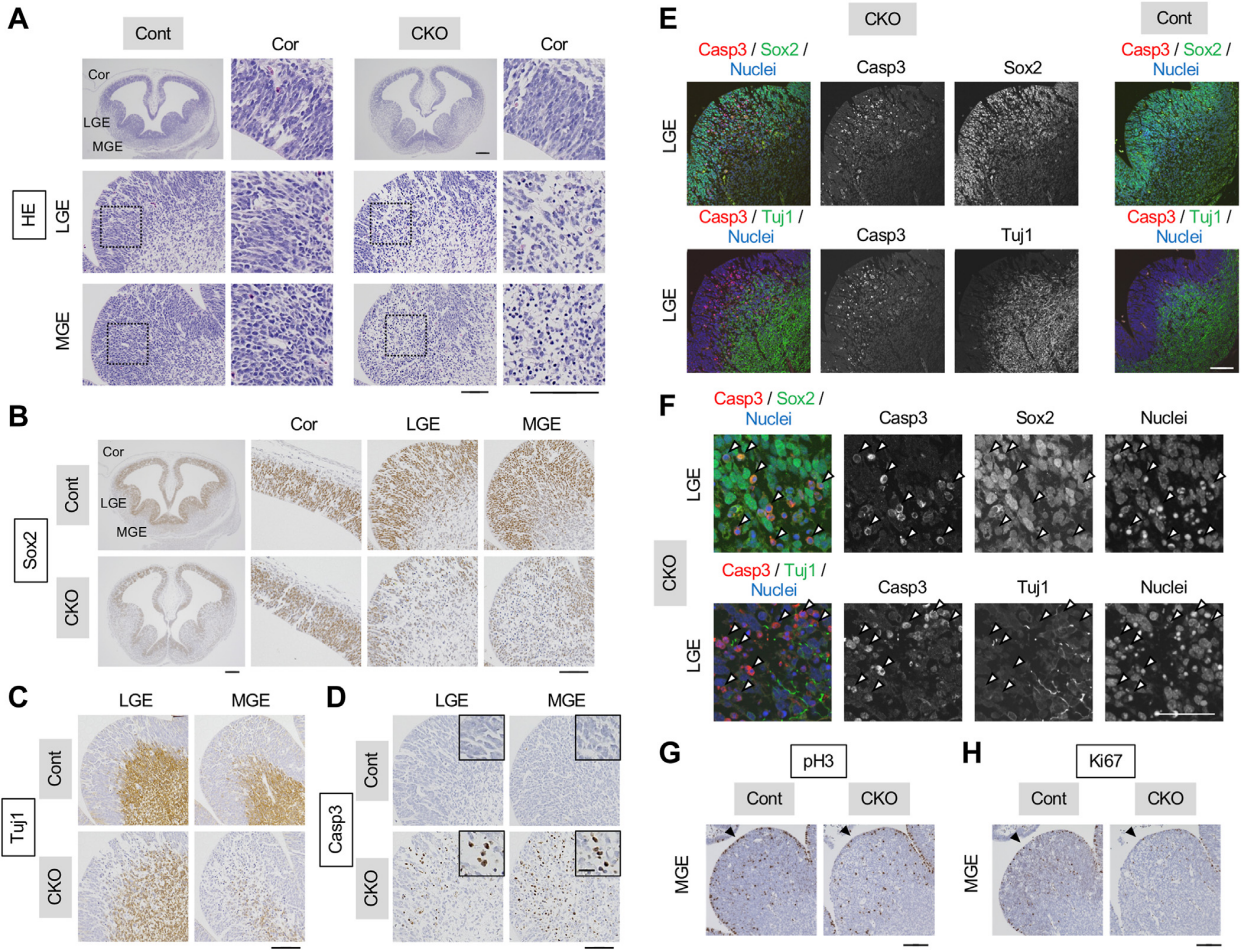
**Severe degeneration and apoptotic cell death of neural progenitors in ganglionic eminences of NF-YA nes-cko mice.***A*, HE staining of coronal sections from NF-YA nes-cko and control mice at E13.5. Cell spears and shrinkage were obvious in the MGE and LGE but lesser in the cortex. *B*–*D*, coronal sections were stained with antibodies (*brown*) for Sox2 (*B*), Tuj1 (*C*), and cleaved caspase-3 (Casp3) (*D*), followed by counter-staining with hematoxylin (*blue*). Note the severe loss of Sox2-positive progenitors and moderate loss of Tuj1-positive neurons in LGE and MGE but not in cortex in association with the presence of a large number of apoptotic cells. Magnified images of boxed regions are displayed in adjacent panels. *E*, coronal sections from NF-YA nes-cko and control mice at E12.5 were costained with cleaved caspase-3 (Casp3) antibody, together with either the antibody for Sox2 or Tuj1, followed by counter-staining with a nuclear marker TOTO-3. *F*, magnified images of the stained sections of NF-YA nes-cko mice. *Arrowheads* indicate the cells positive for Casp3. Note that Casp3 staining accompanied nuclear shrinkage and was highly observed in the cells positive for Sox2-positive progenitors rather than Tuj1-positive neurons. *G* and *H*, coronal sections from NF-YA nes-cko and control mice at E12.5 were immuno-stained with antibodies for cell proliferation markers including phospho-Histone H3 (pH3) (*G*) and Ki67 (*H*). Cells stained with these markers, highly detected at the edge of MGE facing to ventricles, were reduced in nes-cko. The scale bars represent 500 μm (*A* and *B*; whole brain images), 100 μm (*A* and *B*; magnified images, *C*–*E*, *G*, and *H*), 50 μm (*F*), and 20 μm (insets in *D*). Cor, cortex; LGE, lateral ganglionic eminence; MGE, medial ganglionic eminence; NF-Y, nuclear transcription factor-Y; Str, striatum.

The ganglionic eminences (GEs) contain neural progenitors and are major sources of brain inhibitory neurons (2). In MGE, various types of inhibitory neurons are produced and migrate to different brain regions including the pallidum and cortex. In LGE, the striatal medial spiny neurons are produced. The depletion of GEs in the brains of NF-YA nes-cko mice indicates that these regions are also degenerated by NF-YA deletion. To verify this, we analyzed the mice at earlier stages (by E13.5) where no apparent morphological changes were observed (Fig. 1*B*). We first confirmed efficient recombination at E13.5 in the GEs of RNZ mice harboring nestin-cre transgene (Fig. S2*B*). Notably, GEs persisted during the earlier embryonic stages (E12.5–13.5). However, HE staining uncovered GE degeneration characterized by a reduction in cell density and condensed hematoxylin staining in the remaining cells (Figs. 2*A* and S3). The degeneration was more pronounced in MGE than LGE and was almost negligible in the cortex, during these stages, consistent with the genome recombination levels (Fig. S2*B*).

By staining the tissues with several cell markers, we observed a drastic decrease in Sox2 signals, a partial decrease in Tuj1 signals, and the presence of Casp3-positive apoptotic cells in the MGE (Figs. 2, *B*–*D* and S3). These changes are relatively evident in LGE but not present in the cortex at these stages. To identify the cells undergoing apoptosis, we costained the tissue with Casp3 and either Sox2 or Tuj1. As shown in Figure 2*E*, the Casp3 signals were predominantly detected in the apical region of the LGE where the Sox2-positive progenitors were abundant, instead of the basal region containing Tuj1-positive neurons. Detailed analysis showed detection of Casp3 signals in Sox2-positive cells but not Tuj1-positive cells (Fig. 2*F*). Moreover, the cooccurrence of nuclear condensation in these cells further supported the apoptotic cell death in the Sox2-positive progenitors (Fig. 2*F*). Taken together, these data indicate that NF-YA plays a crucial role in maintaining neural progenitors in GEs. Its deletion causes GEs degeneration, leading to the total loss of striatum during brain development.

NF-Y is essential for cell cycle progression in various types of cells, and its inactivation leads to apoptotic cell death possibly associated with cell cycle arrest (5, 6). We checked the proliferation of neural progenitors in GEs by staining them with two cell cycle markers, phospho-histone-H3 (pH3) and Ki67. The cells positive for these markers were partially reduced on the ventricular surface of the MGE in NF-YA nes-cko mice (Figs. 2, *G* and *H* and S3). Quantitative analysis of the surface 20 μm layer revealed that 14.0% of the cells (69 out of 492) were positive for pH3 in nes-cko mice, while 23.7% (161 out of 680) were positive for it in control mice. These data indicate that NF-Y may maintain neural progenitors through the regulation of cell cycle progression and related apoptotic cell death.

We conducted additional costaining experiments on later stage brains to identify the degenerating cells in the cortex by NF-YA deletion. However, apoptotic cells positive for Casp3 were broadly detected in both Sox2-and Tuj1-positive regions in the E14.5 cko cortex (data not shown), as predicted by the single Casp3 staining data (Fig. 1*H*). We speculate that the timing and/or speed of cell migration are different between the cortex and ganglionic eminences, leading to divergences in apoptotic cell distribution. However, because the apoptotic cells are already detected in the VZ, apoptosis or apoptotic pathway could be induced in the progenitors prior to neuronal differentiation and migration in the cortex.

### NF-YA short isoform is primarily expressed in neural progenitors

Our previous research observed no apoptotic cell death in differentiated brain neurons by NF-YA deletion, even though distinct neurodegeneration associated with ER disorganization was induced (8, 9). Therefore, NF-Y may regulate cell maintenance through different mechanisms before and after the neural differentiation. To elucidate the underlying mechanism, we focused on NF-YA splicing (23). We examined whether the neural progenitors express NF-YA short isoform lacking exon 3 (NF-YA-S), as seen in embryonic and hematopoietic stem cells (13, 14, 17, 18), in contrast to the differentiated neurons that predominantly express the NF-YA long isoform (NF-YA-L) (24).

**Figure 3:**
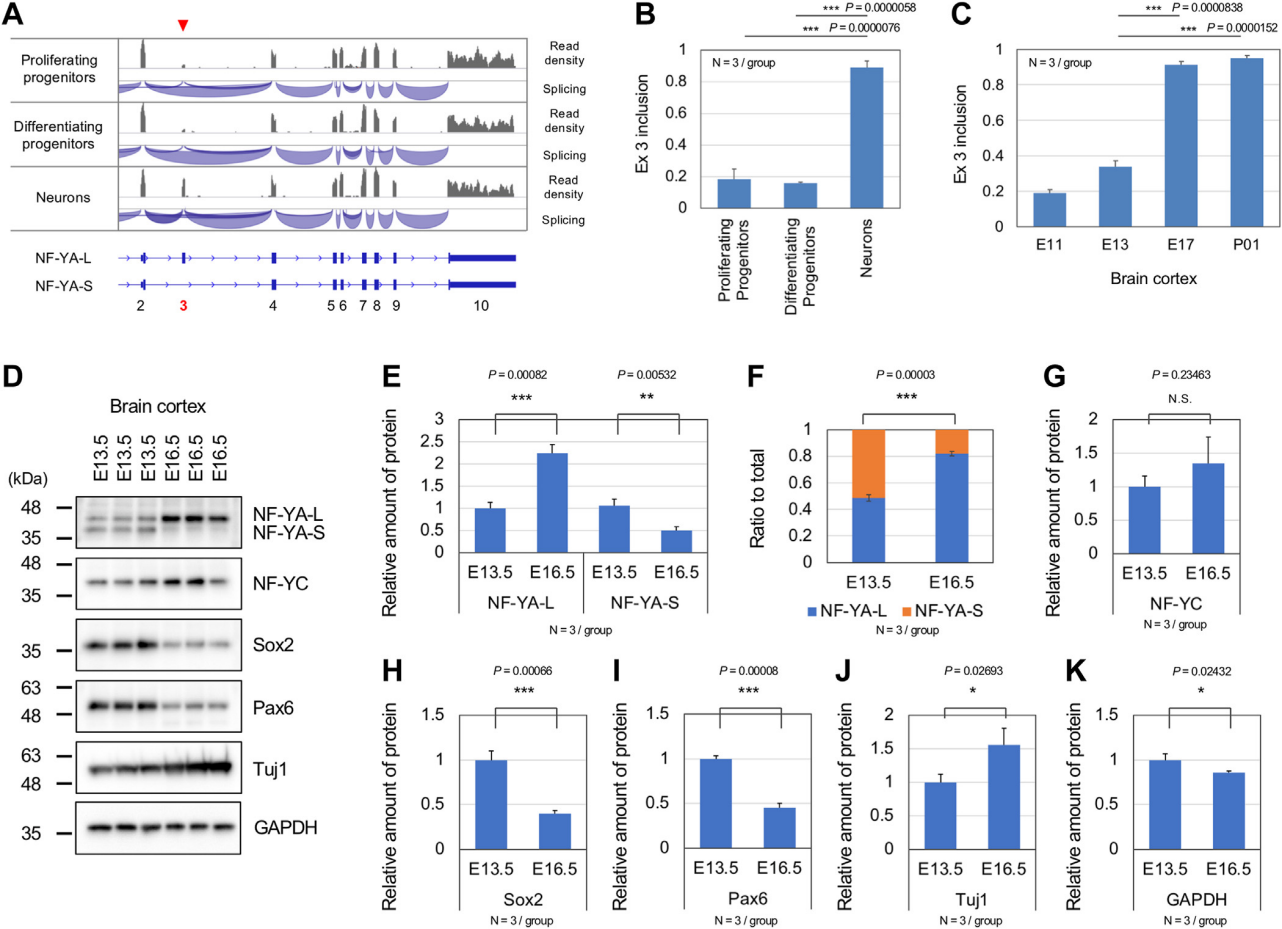
**Predominant expression of NF-YA short isoform in neural progenitors and its replacement with the long isoform during neuronal differentiation.***A*, integrative Genomics Viewer (IGV) shots of RNA-seq data showing read densities and splice junctions of NF-YA in proliferating progenitors, differentiating progenitors, and neurons isolated from brain cortices of mouse embryos. The position of the spliced exon 3 in progenitors is indicated by arrowheads. *B*, estimated ratios of exon 3 inclusion in these three types of cells. Values are means + s.d. of three data and statistically analyzed by one-way ANOVA (∗∗∗*p* < 0.001). *C*, estimated ratio of exon 3 inclusion in mouse brain cortex during development (E13 to P01). Values are means + s.d. of three data and statistically analyzed by one-way ANOVA (∗∗∗*p* < 0.001). *D*, Western blotting of E13.5 and E16.5 mouse brain cortices with antibodies for indicated proteins. *E*, quantification of NF-YA-L and -S proteins. *F*, ratio of the NF-YA-L and -S proteins. *G*–*K*, quantification of other indicated proteins. Values are means + s.d. of three data and statistically analyzed by *t* test (∗*p* < 0.05, ∗∗∗*p* < 0.001). NF-Y, nuclear transcription factor-Y.

We analyzed an RNA-seq dataset of proliferating progenitors (Btg2-/Tubb3-), differentiating progenitors (Btg2+), and neurons (Tubb3+) isolated by cell sorting from the E14.5 embryonic cortices of *Btg2*^*RFP*^*/Tubb3*^*GFP*^ double knock-in mice (25). We observed a significantly lower read density in exon 3 of NF-YA in two progenitors, in contrast to the neurons where the density was largely consistent with the neighboring exons (Fig. 3*A*). By analyzing the number of reads that span the exons, we estimated the inclusion ratio of the exon 3 to be approximately 0.2 in the progenitors and 0.9 in neurons (Fig. 3*B*). Additionally, we examined another RNA-seq dataset of whole cortices isolated from WT mice at various embryonic and postnatal stages (E11 to P1) (26). We found a relatively low exon 3 inclusion (less than 0.4) during earlier stages (E11∼E13) and high inclusion (over 0.9) during later stages (E17 to P1) (Fig. 3*C*). In conjunction with the immunostaining data showing prevalent Sox2 staining in the E13.5 cortex but extensive Tuj1 staining in the E16.5 cortex (Fig. S1), the data provide evidence that NF-YA-S without exon 3 is dominantly expressed in cortical progenitors during early corticogenesis.

We conducted Western blot analysis of the cortices isolated from mouse embryos at E13.5 or E16.5. Anti-NF-YA staining revealed the presence of both short and long isoforms at E13.5 but mainly the long isoform at E16.5 (Fig. 3, *D* and *E*). The ratio of NF-YA-S is altered from 0.51 to 0.18 (Fig. 3*F*). Along with this change, we observed a decrease in Sox2 and Pax6 expression and an increase in Tuj1 expression, while the expression of NF-YC and GAPDH did not show a distinct alteration (Fig. 3, *D*, *G*–*K*). The data align with the RNA-seq splicing data (Fig. 3*C*) and indicate a shift in NF-YA dominance from the short to long isoform during embryonic corticogenesis.

### NF-YA short isoform is co-expressed with cell cycle genes during neurogenesis

**Figure 4:**
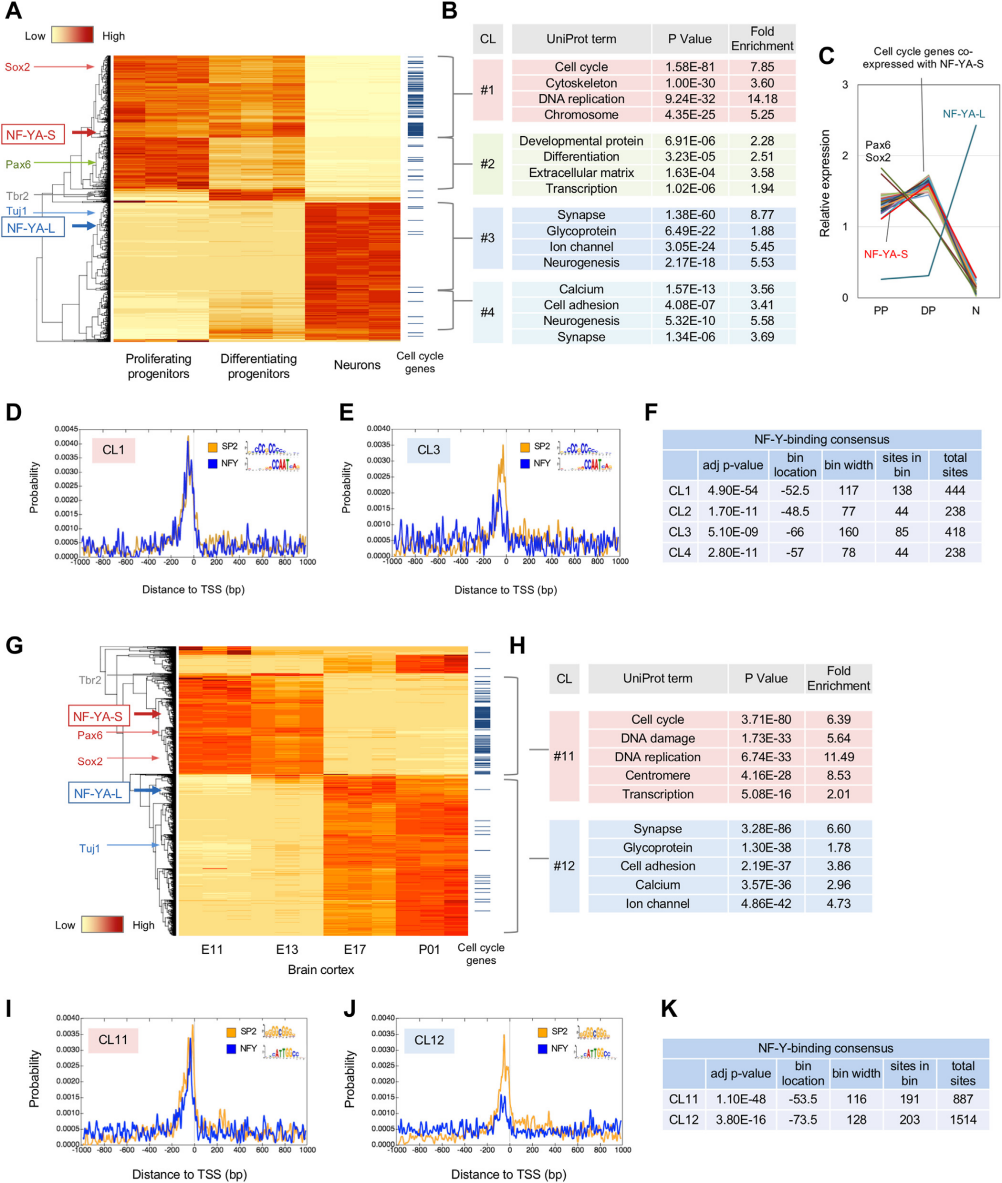
**Co-expression of NF-YA short isoform with cell cycle genes during neuronal differentiation.***A*, heat map and clustering analysis of the genes differentially expressed among proliferating progenitors, differentiating progenitors, and neurons. NF-YA isoforms were included in the analysis. Four major clusters (CL 1–4) were found, with NF-YA-S and -L in CL 1 and CL 3, respectively. Positions of cell markers (*left*) and cell cycle genes (*right*) are indicated. *B*, gene annotation enrichment analysis of the genes in each cluster. Cell cycle–related genes are enriched in the CL 1, whereas genes related to neural functions were enriched in the CL 3. *C*, differential expression of NF-YA isoforms, Sox2, Pax6, and the cell cycle genes coregulated with NF-YA-S. *D* and *E*, CentriMo analysis of TSS ± 1000 bp genomic region of the genes contained in each cluster. The NF-Y-binding CCAAT motif was specifically enriched in the proximal regions of genes in CL 1. *F*, summary of the CentriMo analysis. *G*, heat map and clustering analysis of the genes differentially expressed in mouse cortices during embryonic development (E11 to P01). Two major clusters (CL 11 and 12) were found, with NF-YA-S and -L in CL 11 and 12, respectively. Positions of cell markers (*left*) and cell cycle genes (*right*) are indicated. *H*, gene annotation enrichment analysis of the genes in the clusters. Cell cycle–related genes were enriched in the CL 11, whereas the genes related to neural functions were enriched in the CL 12. *I* and *J*, CentriMo analysis of the TSS ± 1000 bp genomic region of the genes contained in each cluster. The NF-Y-binding CCAAT motif was specifically enriched in proximal regions of genes in CL 11 rather than CL 12. *K*, summary of the CentriMo analysis. NF-Y, nuclear transcription factor-Y.

By analyzing the two RNA-seq datasets, we searched for the genes co-expressing with NF-YA isoforms during neuronal differentiation. Upon examination of the first dataset, we found four major clusters (CL 1–4), with NF-YA-S clustering in CL 1 with enriched cell cycle genes and NF-YA-L clustering in CL 3 with enriched neural genes (Fig. 4, *A* and *B*, Table S1). Several cell cycle genes, such as Ki67, are coregulated with NF-YA-S better than other progenitor markers including Sox2 and Pax6 (Fig. 4*C*). We observed an enrichment of NF-Y-binding CCAAT motifs in the proximal promoter regions of CL 1 genes (Fig. 4, *D*–*F*). In contrast, local enrichment of SP2-boundable GC box was commonly observed for each cluster, indicative of ubiquitous binding of SP transcription factors to the proximal promoters (Fig. 4, *D* and *E*). The second RNA-seq data analysis showed co-clustering of the cell cycle genes and the NF-YA-S isoform in developing cortices (Fig. 4, *G* and *H* and Table S2). Additionally, there was proximal enrichment of NF-Y-binding motifs in the NF-YA-S-containing cluster (CL 11) but not in the NF-YA-L-containing cluster (CL 12) (Fig. 4, *I*–*K*). These data indicate a close association of NF-YA-S with neural progenitor–specific genes, particularly those regulating cell proliferation rather than cell differentiation. The overlap of the NF-Y-binding sites with the GC box indicates the potential cooperation of SP transcription factors with NF-Y in regulating progenitor-specific genes, as previously observed in other contexts (27).

To identify other genes showing alternative splicing during neurogenesis, we analyzed the RNA-seq data with DEXSeq (28), finally identifying 70 differentially expressed exons within 44 transcription-related genes, comprising NF-YA (Fig. S4, Table S3). Conversely, gene expression analysis with EdgeR (29) identified 207 transcription-related genes showing differential expression, with slight overlap among the aforementioned 44 genes (Fig. S4, Table S4). Thus, alternative splicing could serve as a regulatory mechanism for certain transcription-related genes during the process of neuronal differentiation.

### NF-Y targets cell cycle genes during early corticogenesis

**Figure 5:**
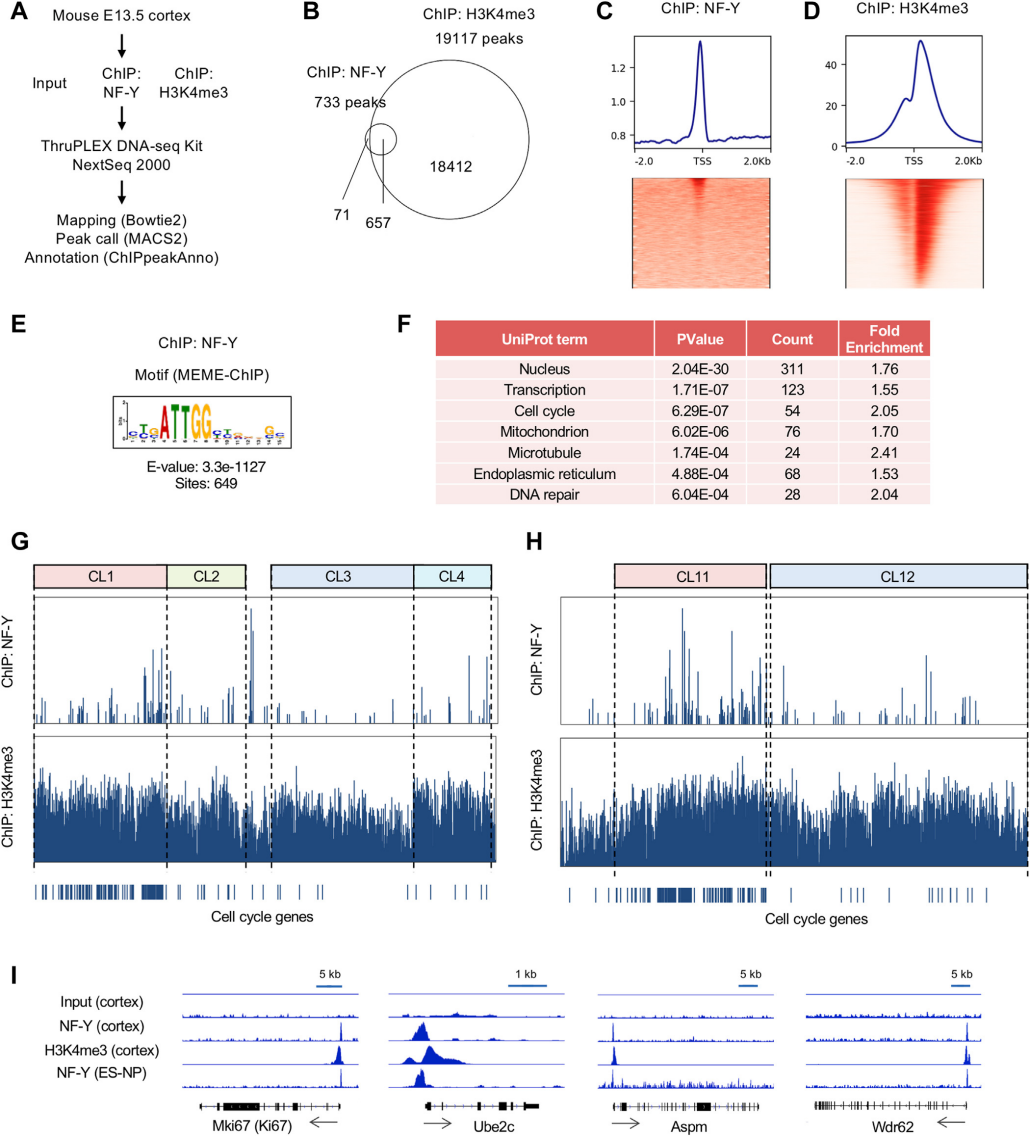
**Identification of NF-Y-target genes at the early stage of corticogenesis.***A*, scheme of the ChIP-seq of NF-Y and H3K4me3 from E13.5 mouse brain cortices. *B*, peaks identified by NF-Y-ChIP and H3K4me3-ChIP and their overlaps. *C* and *D*, heatmap and density plots of ChIP peaks of NF-Y (*C*) and H3K4me3 (*D*) within ± 2 kb of TSS. *E*, NF-Y-binding motif identified by MEME-ChIP. *F*, gene ontology enrichment analysis of the NF-Y-target genes identified by the ChIP-seq. *G* and *H*, ChIP-peaks of NF-Y and H3K4me3 in differentially expressed genes during neurogenesis (*G*) and corticogenesis (*H*) clustered in Figure 4. *I*, IGV shots of several NF-Y-binding cell cycle genes. Peaks for inputs, NF-Y-ChIP, and H3K4me3-ChIP from E13.5 cortices (cortex), as well as peaks for NF-Y-ChIP from ES-derived neural progenitors (ES-NP), are shown. NF-Y, nuclear transcription factor-Y.

To identify the target genes of NF-Y in early corticogenesis, we conducted chromatin immunoprecipitation (ChIP) on E13.5 cortices. We tested two antibodies, NF-YA (YA1-3) and NF-YC (YC5-3), reactive to the endogenous proteins by Western blotting (Fig. 3*D*), for ChIP. However, only YC5-3 was able to precipitate known NF-Y-binding promoters (data not shown) and therefore was utilized for NF-Y-ChIP. An antibody for histone H3 tri-methyl Lys4 (H3K4me3), a chromatin mark of active transcription (30), was also employed as a positive control, while an input for ChIP served as a negative control. The ChIP-seq procedure is briefly summarized in Figure 5*A*. NF-Y-ChIP identified 733 binding regions, of which 90% (657) were located within H3K4me3-positive genome regions (19,117 regions) (Fig. 5*B*). The density plot revealed a single peak corresponding to the binding of NF-Y to proximal promoter regions (Fig. 5*C*), whereas the H3K4me3-ChIP displayed typical double peaks (Fig. 5*D*). The NF-Y-ChIP showed enrichment of the NF-Y-binding motif (ATTGG/CCAAT) (Fig. 5*E*). Gene annotation identified 969 potential NF-Y-target genes associated with various cellular functions, including cell cycle genes (Fig. 5*F*, Tables S5 and S6). Targets of both NF-YA isoforms may be present based on the Western blot data (Fig. 3*D*), and we mapped them to the gene clustering data to classify them. The NF-Y-ChIP peaks were relatively enriched in the clusters containing NF-YA-S and cell cycle genes (48% in CL 1 and 65% in CL 11) (Fig. 5, *G* and *H*). Conversely, the H3K4me3-ChIP peaks were distributed throughout clusters (Fig. 5, *G* and *H*, Table S7). ChIP peaks for several cell cycle genes, such as Mki67 (Ki67), Ube2c, Aspm, and Wdr62, are displayed in Figure 5*I*. We also included the published NF-Y-ChIP-seq data for embryonic stem–derived neural progenitors (31) and peaks similar to those in our data were confirmed (Fig. 5*I*). These data suggest that NF-Y preferentially binds to the promoters of cell cycle genes expressed in neural progenitors.

Given the dominant expression of NF-YA-S in the progenitors and its coregulation with cell cycle genes during development, NF-YA-S could be the critical regulator of these genes. Immunostaining has already verified the reduction of Ki67 (Figs. 2*H* and S3), and we also examined the expressions of Ube2c and Aspm through immunostaining. Their staining was relatively enriched in the regions facing the ventricles in the cortex and GEs of the E12.5 brain. However, it was preferentially reduced in the MGE of the NF-YA nes-cko brain (Fig. S5), which is consistent with the high cre expression (Fig. S2) and severe neurodegeneration in the MGE (Fig. S3). Due to the region- and stage-specific deletion of NF-YA by cre in embryonic brains and the subsequent progressive cell loss accompanied by apoptosis, analyzing target gene expressions using bulk brain tissues may be difficult and therefore was not attempted in this study. However, the data suggest that the deletion of NF-YA in the brain may impact the expression of certain NF-Y targets.

Relatively fewer NF-Y-ChIP peaks in the clusters with NF-YA-L (CL 3 and 12) imply that NF-YA-L may not regulate neuron-specific genes through the binding to their proximal promoters. Rather, NF-Y could bind distal regions (enhancers) to regulate neuron-specific genes as observed in differentiated embryonic stem cells (19). Therefore, we extended the genomic region for peak annotation to 50 kb from the TSS. Upon plotting the distance of the gene-annotated peaks from the TSS, we observed that 90% of the peaks were located within 1 kb for genes in the clusters containing NF-YA-S (CL 1 and 11). In contrast, around 40% of peaks remain located in distal regions beyond 1 kb for genes in the NF-YA-L-containing clusters (Fig. S6). Therefore, the regulation mechanism may vary for neuron-specific genes through NF-YA-L. Nonetheless, additional examination is necessary to draw a final conclusion.

### Forced expression of NF-YA long isoform in neural progenitors resulted in a decrease in the number of neurons

**Figure 6:**
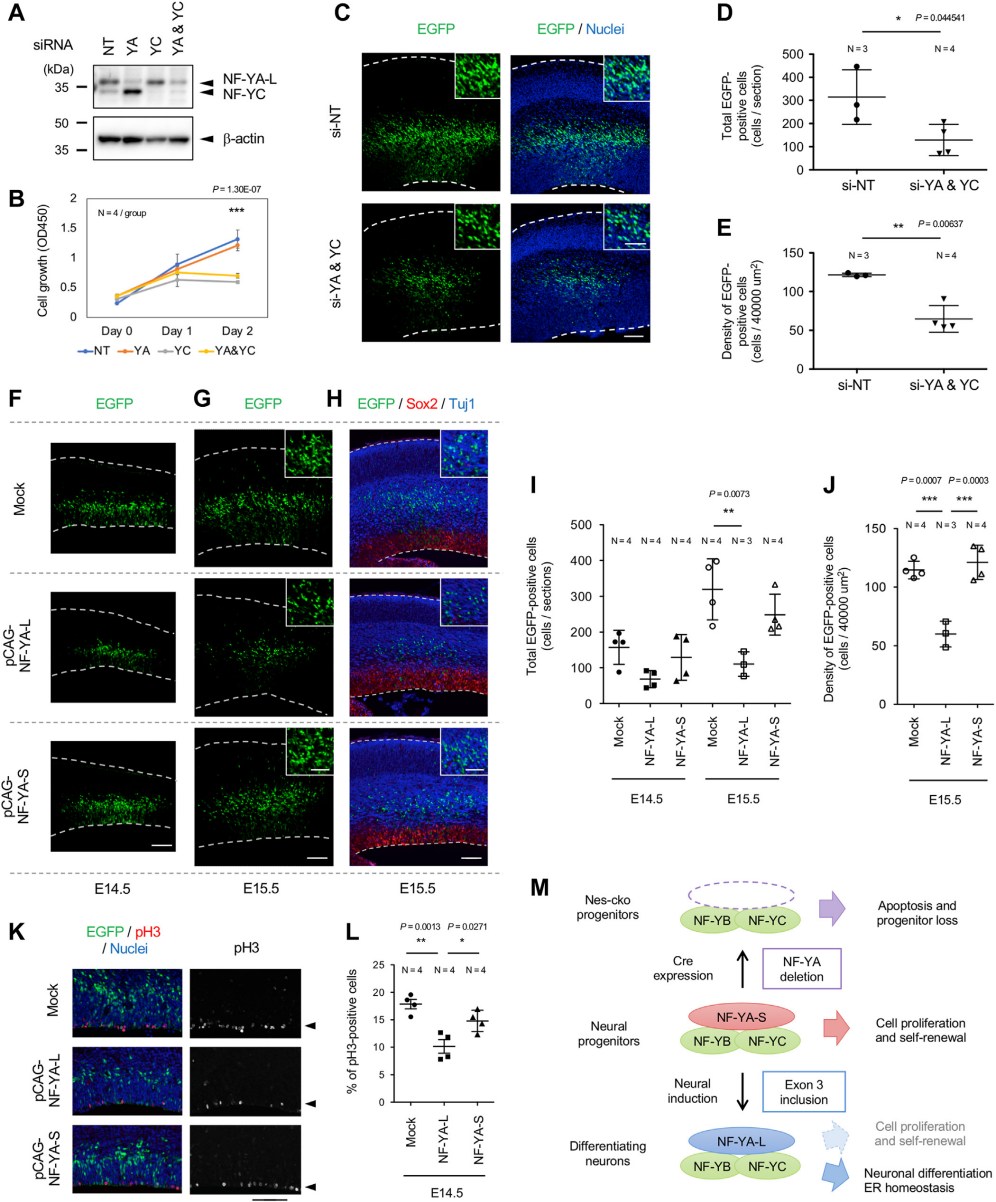
**Knockdown of NF-Y or overexpression of NF-YA-L in cortical progenitors resulted in a decline in neurons.***A*, Western blotting of N2a cells transfected with siRNAs for non-targeting (NT), NF-YA, NF-YC, and a mix of the latter two. Blots were stained with mixed anti-NF-YA and -YC antibodies (*upper*) and β-actin (*bottom*). *B*, cell growth assay of siRNA-transfected N2a cells. *C*, mouse embryos at E13.5 were subjected to IUE with NT siRNA or NF-YA and NF-YC mixed siRNAs, together with pCAG-EGFP for cell labeling. The mouse brains were fixed at 2 days (E15.5) after the electroporation, followed by cryo-sectioning and nuclear (DAPI) staining. *D*, total number of EGFP-positive cells per section. *E*, density of EGFP-positive cells in the area of 40,000 μm^2^. *F* and *G*, mouse embryos at E13.5 were subjected to IUE with pCAG empty vector (mock), pCAG-NF-YA-L, or pCAG-NF-YA-S, together with pCAG-EGFP for cell labeling. The mouse brains were fixed at 1 day (E14.5) (*F*) or 2 days (E15.5) (*G*) after the electroporation, followed by cryo-sectioning. *H*, brain sections of E15.5 embryos were stained with the progenitor marker Sox2 and the neuronal marker Tuj1. *I*, total number of EGFP-positive cells per section. *J*, density of EGFP-positive cells in the area of 40,000 μm^2^. *K*, brain sections of E14.5-electroporated embryos were stained with a cell proliferation marker phospho-Histone H3 (pH3; *red*) together with a nuclear marker TOTO-3 (*blue*). *L*, cells positive for pH3 or TOTO-3 were counted in the region with a 20 μm width from the edge to ventricle, and the population of pH3-positive cells per total cells was quantified. For each experiment, at least three samples/mice were analyzed and means + s.d. are indicated. Statistical analysis was performed by one-way ANOVA followed by Tukey's post hoc tests (∗*p* < 0.05, ∗∗*p* < 0.01, ∗∗∗*p* < 0.001). Scale bars are 100 μm (*C*, *F*, *G*, *H*, and *K*) and 50 μm (insets in *C*, *G*, and *H*). *M*, schematic model of the role of NF-Y in neural progenitor maintenance and differentiation. In neural progenitors, NF-YA-S isoform is dominantly expressed and forms a ternary complex with NF-YB and NF-YC to promote progenitor proliferation and self-renewal. Deletion of NF-YA-S inactivates NF-Y, resulting in decreased proliferation and induction of apoptosis in the progenitors. Upon neural induction, the expression of NF-YA-L isoform containing exon 3 becomes dominant, leading to a shift in the function of NF-Y from cell progression to maintaining ER homeostasis and/or promoting neuronal differentiation. ER, endoplasmic reticulum; NF-Y, nuclear transcription factor-Y.

To elucidate the significance of the NF-YA isoform switch during neuronal differentiation, we employed *in utero* electroporation (IUE). Initially, we conducted NF-Y knockdown in neural progenitors by IUE to check the effect of NF-Y suppression through this method. The siRNA effect was assessed in neuro2a mouse neuroblastoma cells that exclusively express NF-YA-L isoform (24). As shown in Figure 6*A*, the siRNAs specifically knocked down NF-YA and NF-YC, compared to non-targeting (NT) control, while NF-YA siRNA caused a compensatory upregulation of NF-YC, as described previously (7, 8). Mixed siRNA transfection caused significant downregulation of both genes (Fig. 6*A*). Either NF-YC siRNA alone or mixed siRNAs was effective in suppressing the growth of neuro2a cells (Fig. 6*B*). However, NF-YA siRNA alone was ineffective, possibly due to insufficient downregulation of NF-YA and/or compensatory upregulation of NF-YC.

We then performed IUE on E13.5 embryos using either the NT siRNA or mixed NF-YA and NF-YC siRNAs in conjunction with pCAG-EGFP to monitor the transfected cells. The embryonic brains were fixed 2 days after the IUE, and those with higher EGFP fluorescence were chosen for cryo-sectioning. Three to four sections (100 μm intervals) consisting of the highest number of EGFP-positive cells were quantitatively analyzed. As shown in Figure 6*C*, NF-YA and NF-YC knockdown did not affect cell migration or differentiation. The majority of EGFP-labeled cells were located outside of the VZ but within both subventricular and intermediate zones after IUE of NT siRNA or mixed siRNAs for NF-YA and NF-YC. Conversely, the number of EGFP-positive cells was reduced by the mixed siRNAs (Fig. 6*C*). Quantification analysis revealed around a 50% reduction in cell number and density (Fig. 6, *D* and *E*), which may reflect the reduced proliferation of progenitors. No clear induction of apoptosis was evident with NF-YA and NF-YC knockdown, indicated by the absence of distinct Casp3 staining (Fig. S7*A*). This could be due to incomplete gene downregulation by the siRNAs. Additionally, active proliferation and differentiation of the progenitors may contribute to the reduction of the knockdown effect, as the cells were rapidly expanded and differentiated within 2 days after IUE (Fig. 6, *C*, *F* and *G*). Nonetheless, moderate effects were induced by NF-Y suppression through the knockdown.

We next overexpressed the NF-YA isoforms in cortical progenitors *via* IUE on E13.5 embryos with the NF-YA-S or -L expression vectors along with pCAG-EGFP. We performed cryo-sectioning and quantitative analysis as described above. NF-YA-L overexpression resulted in a low number of EGFP-labeled cells (Fig. 6, *F* and *G*). The number and density of EGFP-positive cells decreased by over 50% at 2 days (E15.5) after the IUE compared to the mock (Fig. 6, *I* and *J*). This effect is exclusive to NF-YA-L, as overexpression of NF-YA-S did not affect the count or density of EGFP-positive cells (Fig. 6, *F*–*J*). On the contrary, radial migration and neuronal differentiation may remain unaffected by NF-YA-L expression, as the comparable migration of the EGFP-positive cells to the mock-transfected cells (Fig. 6, *F* and *G*). Additionally, the EGFP-positive cells were negative for Sox2 while codistributing with Tuj1 at E15.5 (Fig. 6*H*). Staining of the tissues with Casp3 revealed no significant increase in apoptotic cells by NF-YA-L expression (Fig. S7*B*). By using transfected neuro2a cells, we confirmed that the overexpression of NF-YA isoforms did not affect EGFP expression or cell viability (Fig. S8). Finally, we assessed progenitor proliferation by staining the E14.5 brains with phospho-Histone H3 (pH3), showing a significant reduction (approximately 40%) in pH3-positive cells upon the introduction of NF-YA-L (Fig. 6, *K* and *L*). These data suggest that exogenous NF-YA-L expression may decrease progenitor proliferation during cortical development.

## Discussion

To elucidate the role of NF-Y in neural progenitors, we here generated NF-YA conditional deletion mice through the nestin-cre transgene. These mice exhibited embryonic (or neonatal) lethality and displayed significant defects in their brain structures during embryogenesis. Progenitors in the VZ of cortex declined progressively, accompanied by a reduction in neurons and the induction of apoptosis. Similarly, neural progenitors of ganglionic eminences underwent progressive reduction and apoptotic death, resulting in the loss of the striatum in the brain. Further analysis revealed dynamic splicing of NF-YA isoforms, from the short (NF-YA-S) in neural progenitors to the long (NF-YA-L) in neurons, which correlated with the downregulation of cell cycle genes. ChIP-seq analysis revealed that NF-Y targets the proximal promoters of cell cycle genes to regulate their expression during early corticogenesis. Moreover, forced expression of NF-YA-L in neural progenitors by IUE resulted in a decline in neurons, possibly by suppressing progenitor proliferation. These findings suggest that the NF-Y complex containing NF-YA-S is pivotal in the maintenance and progression of neural progenitors, and the switching to NF-YA-L may be the critical step in modulating progenitor proliferation by NF-Y (Fig. 6*M*).

NF-YA-S lacks exon 3, encoding 28 amino acids within the glutamine-rich transcriptional activation domain (23). While NF-YA-S and -L isoforms have comparable activities in DNA binding and gene transactivation, they exhibit distinct tissue-specific expression; NF-YA-S is prevalent in the thymus and spleen while NF-YA-L is dominant in the liver and brain (23). We have found, for the first time, that NF-YA-S is predominantly expressed in neural progenitors and is crucial for their maintenance. This is consistent with observations in other types of stem cells (13, 14, 17, 18), highlighting the significance of NF-YA-S in the maintenance of stem/progenitor cells. Consistently, NF-Y targets comprise a number of genes specific to neural progenitors, including cell cycle genes. Additionally, we observed its switching to the -L isoform during neural differentiation. The short-to-long switch has been reported in ES cells during differentiation by retinoic acid (17, 18). Conversely, long-to-short switch has been reported in breast cancer cells (32). The predominance of NF-YA-S expression is further observed in other types of cancer, including lung adenocarcinoma, lung squamous cell carcinomas, and prostate cancers (33, 34, 35). Notably, in prostate cancers, NF-YA-S overexpression enhances cell proliferation while NF-YA-L increases cell motility (35), suggesting differential roles of NF-YA isoforms in cancer progression. Therefore, the variation in NF-YA isoforms is closely associated with cell proliferation and differentiation states, with NF-YA-S playing a specific role in cell proliferation.

Many cell cycle genes have been identified as targets of NF-Y in various proliferating cells (19, 24). Our ChIP-seq analysis further revealed a relative enrichment of genes linked to mitosis or cell division (Table S6). These include Ube2c (36), Ki67 (37), Aspm (38), and Wdr62 (39), all of which play roles in M phase regulation. For instance, Aspm and Wdr62 are linked to autosomal recessive microcephaly and localize at mitotic spindle poles to regulate spindle orientation during mitosis (39). As the cells in M phase, positive for phospho-histone H3, decreased in the NF-YA cko brains (Fig, 2G, S3), the M phase entry and/or progression can be regulated by NF-Y through regulating these genes. Studying each target gene may provide a deeper understanding of the mechanism underlying stem cell progression by NF-Y.

Exogenously expressed NF-YA-L may counteract NF-YA-S in regulating progenitor proliferation and maintenance *via* NF-Y. Additionally, NF-YA-L binding to distal enhancers may contribute to the loss of proliferative activity by inducing the expression of neuron-specific genes, as we observed preferential binding of NF-Y to distal regions in the genes co-expressed with NF-YA-L. Furthermore, NF-YA-L binds to the proximal promoters of ubiquitous proteins, such as ER chaperones, to regulate cellular homeostasis and promote neuronal survival (8, 9, 24). Therefore, isoform switch may induce transcriptomic alterations at multiple points to regulate neuronal differentiation and its maintenance (Fig. 6*M*).

In contrast to other stem/progenitors, including the neural progenitors shown here, myoblasts predominantly express NF-YA-L; however, the expression is downregulated during differentiation into muscle cells (15, 16). Overexpression of the two NF-YA isoforms differentially affects myoblast fate: NF-YA-S promotes cell proliferation, while NF-YA-L boosts cell differentiation (16). Similarly, NF-YA-L is predominantly expressed in satellite cells, the stem cell population of adult skeletal muscle (40). Its conditional knockout resulted in impaired differentiation of satellite cells and delayed muscle regeneration after injury. Notably, the knockout induces upregulation of cell cycle genes and downregulation of genes involved in muscle differentiation. These studies further highlight the essential role of the long isoform in cell differentiation as opposed to cell proliferation.

In summary, we have elucidated the role of NF-Y in maintaining neural progenitors in mouse embryos and suggested the potential significance of the isoform switching of NF-YA in regulating cell proliferation. Due to the severe and global degeneration resulting from NF-YA deletion, we propose that NF-Y is a general regulator crucial for the maintenance of multiple neural progenitors that generate diverse neuron types. Our data present a novel way to regulate progenitor maintenance through gene splicing. In addition to NF-YA, we identified other transcriptional regulators that alter splicing without altering their gene expression during neuronal differentiation. Further investigation of these factors in future studies may provide more insight into the mechanism of neuronal differentiation.

## Experimental procedures

### Mice

The mouse experiments were conducted in compliance with the guidelines and regulations of RIKEN, Doshisha University, and Niigata University, following approval by the animal experiment committees at these institutions. The NF-YA flox mice (12) were maintained on a C57BL6 (B6) background by mating with female B6 mice. The neural progenitor–specific Nestin-cre (nes-cre) transgenic mice expressing cre recombinase under the control of the mouse nestin gene promoter and second intronic neural enhance (21) were generously provided by Dr Okano (Keio University) and Dr Mizushima (The University of Tokyo) and were maintained on a B6 background. For generation of neural progenitor–specific NF-YA deletion mice (NF-YA nes-cko mice) with the genotype of flox/flox; nes-cre, NF-YA flox/+; nes-cre mice were crossed with NF-YA flox/flox mice. Other littermates with different genotypes (NF-YA flox/+; nes-cre, NF-YA flox/flox, or NF-YA flox/+) were used as control mice due to their lack of apparent phenotypes. RNZ (ROSA26-loxP-STOP-loxP-nlsLacZ) mice (22), expressing LacZ in nuclei upon cre-mediated recombination, were generously provided by Dr Itohara (RIKEN BSI). The primer sets used for genotyping were described previously (8).

### Antibodies

Primary antibodies for Sox2 (rabbit; ab97959), Sox2 (mouse; ab79351), Ki67 (rabbit; ab16667), Tbr2 (rabbit; ab23345), LacZ (chick; ab9361), Histone H3 trimethyl K4 (H3K4me3) (rabbit; ab8580), and GFP (chick; ab13970) were from abcam; Pax6 (rabbit; PD022) was from MBL; phospho-Histone H3 (Ser10) (rabbit; 06-570) was from Millipore; Cleaved Caspase-3 (rabbit; 9664S) was from Cell Signaling Technology; Beta-III tubulin (Tuj1) (mouse; MAB1195) was from R&D systems; GAPDH (mouse;016-25523) was from Wako, Ube2c (rabbit; 12134-2-AP) and ASPM (rabbit; 26223-1-AP) were from Proteintech. Antibodies for NF-YA (YA1-3) and NF-YC (YC5-3) were described previously (7).

### Histological analysis of NF-YA nes-cko mice

NF-YA nes-cko and control mice were deeply anesthetized and sacrificed. After isolating the embryos, their skulls containing brains were fixed with 4% PFA in PBS overnight. The brains were isolated, dehydrated, and embedded in paraffin. For embryos at E12.5, whole skulls were used for embedding. Coronal sections at 5 μm thick were prepared from the paraffin blocks and deparaffinized. HE staining was performed using Carazzi's hematoxylin and eosin. For immunohistochemistry, the sections were autoclaved in 10 mM citrate buffer (pH 6.0) at 120 °C for 5 min, followed by staining with antibodies as described previously (41). After the staining, the sections were briefly counter-stained with Mayer's hematoxylin. For immunofluorescence analysis, the sections were stained as described previously (9), and nuclei were visualized with TOTO-3 (Thermo Fisher). Images were obtained using a Keyence microscope (BZ-X710) or an Olympus confocal system (FV1000).

### Embryo cortices isolation and western blotting

Cerebral cortices were isolated from E13.5 and E16.5 B6 embryos and lysed with a lysis buffer containing 50 mM Tris–HCl, pH 8.8 and 1% SDS. After measuring protein concentration using the BCA method, the lysates were mixed with SDS sample buffer and 10 μg of them were subjected to SDS-PAGE followed by Western blotting as described previously (42). Chemiluminescent signals were obtained and quantified using ImageQuant LAS-4000 (GE) or Multi Imager II (IEDA).

### RNA-seq data analysis

To analyze the RNA-seq data of cortical progenitors/neurons isolated from E14.5 mouse embryos by sorting (GSE51606) (25) or those of cortices of mice at ages of E11, 13, 17 or P1 (GSE154677) (26), SRA data were retrieved from GEO/SRA or DDBJ database sites and extracted using SRAToolkit. After a quality check using FastQC, the reads underwent quality control using Trimmomatic (43) and PRINSEQ (44). Trimmed reads were then aligned to the mouse (mm10) genome using HISAT2 (45), and obtained SAM format files were converted to BAM format files using Samtools (46). Read densities and splice junctions were visualized using Integrative Genomics Viewer (47). To examine exon 3 inclusion, reads spanning exons 2-3, 3-4, or 2-4 were manually counted, and the sum of the former two was divided by the sum of total counts where counts for 2-4 were doubled. For gene expression analysis, reads on the genes were counted using featureCounts (48), and differentially expressed genes were identified using an R package edgeR (29). For exon expression analysis, reads on the exons were counted using featureCounts (48) using an annotation file generated using dexseq_prepare_annotation2.py (28) and then differentially expressed exons were identified using an R package DEXSeq (28). The generation of heatmaps and clustering of samples/genes were performed using an R package heatmap. Gene ontology enrichment analysis was performed using the DAVID bioinformatics database (49). Locally enriched TF-binding motifs around the TSSs of differentially expressed genes were identified using CentriMo (50) as described previously (24).

### ChIP-seq

For ChIP, cerebral cortices of E13.5 embryos were minced with blades and fixed with 1% formaldehyde (Pierce) in PBS at room temperature for 10 min. After adding 10% v/v of 1M glycine/PBS, the tissues were washed twice with PBS and then lysed with SDS buffer containing 1% SDS, 10 mM EDTA, 50 mM Tris, pH 8.0, 1x cOmplete (SIGMA). The lysates were sonicated 45 times for 30 s at 30 s intervals using a BioruptorII (BM bio) and clarified by centrifugation at 20,000*g* for 10 min. The supernatant was diluted 10-fold with ChIP dilution buffer containing 0.01% SDS, 1.1% Triton X-100, 1.2 mM EDTA, 16.7 mM Tris-HCl, pH 8.0, 167 mM NaCl, 1x cOmplete. The dilute (input) was incubated with antibodies for NF-YC (YC5-3) or H3K4me3 overnight at 4 °C and then incubated with Dynabeads Protein G for 1 h. The beads were sequentially washed with low salt buffer (0.1% SDS, 1% Triton X-100, 2 mM EDTA, 20 mM Tris-HCl, pH 8.0, 150 mM NaCl), high salt buffer (0.1% SDS, 1% Triton X-100, 2 mM EDTA, 20 mM Tris–HCl, pH 8.0, 500 mM NaCl), LiCl buffer (0.25 M LiCl, 1% NP-40, 1% deoxycholate, 1 mM EDTA, 10 mM Tris, pH 8.0), and TE (10 mM Tris–HCl, pH 8.0, 0.1 mM EDTA). The beads were incubated with elution buffer (1%SDS, 0.1 M NaHCO_3_) for 30 min. The input was also diluted with the elution buffer. After adding NaCl (final 0.2 M), the ChIP samples and input were incubated at 65 °C for overnight, followed by the addition of EDTA (final 10 mM), Tris HCl, pH 6.8 (final 40 mM), 20 μg proteinase K, and incubation at 45 °C for 1 h. After phenol/chloroform extraction and ethanol precipitation, the ChIP and input DNAs were dissolved in nuclease-free water.

For sequencing, libraries of the input, NF-Y-ChIP and H3K4me3-ChIP DNAs were generated using a ThruPLEX DNA-seq Kit (Takara) according to the manufacture's protocol. The libraries were quality-checked using an Agilent 4200 TapeStation with High Sensitivity D5000 and quantified using a Bio-rad CFX Connect with KAPA HiFi HS ReadyMix (NIPPON Genetics). Subsequently, they were sequenced using an Illumina NextSeq 2000 with P1 regent (paired-end; 50 cycles each).

### ChIP-seq data analysis

The sequence reads (fastq files) were quality-checked using FastQC and underwent quality control with Trimmomatic (43). Trimmed reads were then aligned to the mouse (mm10) genome using Bowtie 2 (51), and the resulting SAM format files were converted to BAM format files using Samtools (46). Read densities were visualized using the Integrative Genomics Viewer (47). ChIP peaks were called using MACS2 (52) with a significant cut-off of *p* < 0.001 (NF-Y-ChIP) or *q* < 0.05 (H3K4me3-ChIP). ChIP peaks were annotated based on their locations using ChIPpeakAnno (53), and genes containing peaks inside or within 5 kb upstream of those were considered as NF-Y or H3K4me3 targets. For the annotation of NF-Y-ChIP peaks on distal regions, peaks within the upstream 50 kb to genes were selected, and if multiple genes were annotated, the closest genes were considered potential targets regulated by the distal regions. Gene ontology enrichment analysis was performed using the DAVID bioinformatics database (49). To visualize the ChIP-peaks by heatmap, bigwig files were first generated using UCSC utilities; bedSort for sorting the bedgraph files and bedGraphToBigWig for conversion to bigwig files. Then, deepTools was used to plot the heatmap of the NF-Y- and H3K4me3-ChIP peaks around TSS (54). For NF-YA-ChIP-seq data of mouse neural progenitors (GSE25532) (31), Sequence Read Archive (SRA) data were retrieved from GEO/SRA database site and extracted using SRAToolkit. The read sequence data were analyzed as described above. Motif discovery was performed using MEME-ChIP (55).

### *In utero* electroporation and immunofluorescence analysis

siRNA (siGENOME) oligos for Non-Targeting Pool #2 (D-001206-14-05), mouse NF-YA (D-065522-04-0005), and mouse NF-YC (M-060374-01-0005) were purchased from Dharmacon. A plasmid vector for full-length mouse NF-YA (NF-YA-L) was described previously (7) and that for its short isoform lacking exon 3 (NF-YA-S) was generated by a PCR-based method using two primers: CAGATTCAGCAGCAGGTCCAGGGGCAGCCG and CGGCTGCCCCTGGACCTGCTGCTGAATCTG. cDNAs for these NF-YA isoforms were inserted into the pCAG-neo vector to generate their expression vectors (pCAG-NF-YA-L and pCAG-NF-YA-S). IUE was performed as described previously (56). Briefly, pregnant ICR mice were deeply anesthetized with a mixed anesthetic (medetomidine, midazolam, and butorphanol), and siRNA oligos or plasmid DNAs (pCAG-neo, pCAG-NF-YA-L, or pCAG-NF-YA-S) were co-injected with pCAG-EGFP into brain ventricles of E13.5 embryos and were introduced to neural progenitors by electroporation. The mouse embryos were sampled 1 or 2 days after the IUE, and isolated brains were fixed with 4% paraformaldehyde/PBS for 2 h and then cryoprotected with 30% sucrose/PBS for overnight. During this process, the brains with weaker EGFP signals under a fluorescence microscope were discarded, and all of the brains showing higher EGFP fluorescence were used for cryo-sectioning to obtain serial sections (10 μm thick). We aimed to obtain all of the sections containing a certain number of EGFP-positive cells (∼100 sections/a frozen block). Immunofluorescence analysis was performed as above, and nuclei were stained with DAPI or TOTO-3. Images were obtained on a Keyence microscope (BZ-X710) or an Olympus confocal system (FV1000). For quantification of cell number, sections at 100 μm intervals were selected to avoid cell overlapping, and EGFP-positive cells were counted using ImageJ software (57). For the quantification of cell density, an area of 40,000 μm^2^ was selected manually, and EGFP-positive cells were counted as above.

### Neuro2a cell transfection, Western blotting, and cell viability assay

Neuro2a mouse neuroblastoma cells, generously provided by Dr Iwatsubo (Tokyo University) (58), were maintained in DMEM supplemented with 10% FBS and penicillin-streptomycin in an atmosphere containing 5% CO_2_. The cell lines were regularly checked to be free from *mycoplasma*. For knockdown, the cells were transfected with siRNA oligos using RNAiMAX (Invitrogen) according to the manufacture's protocol. For overexpression, the cells were transfected with pCAG-EGFP along with pCAG-neo, pCAG-NF-YA-L, or pCAG-NF-YA-S using lipofectamine 2000 (Invitrogen) according to the manufacture's protocol. For Western blotting, the cells cultured for 48 h were then subjected to SDS-PAGE followed by Western blotting as described previously (42). Chemiluminescent signals were obtained and quantified using an ImageQuant LAS-4000 (GE) or a Multi Imager II (IEDA). For the cell growth assay, the transfected cells were re-seeded and grown on a 96 well plate and then incubated with Cell Counting Kit-8 (DOJINDO) for 1 h, followed by the measurement of absorption at 450 nm using a SpectraMax iD3 (Molecular Devices). For the cell death assay, the transfected cells were incubated with culture media containing 10 μm propidium iodide and 10 μg/ml Hoechst 33342 for 10 min. Fluorescence cell images (ultraviolet and red channels) in living cells were automatically obtained and analyzed using a Thermo Scientific CellInsight NTX image analyzer. Over 3000 cells were analyzed for each well, and the populations of PI-stained dead cells in the Hoechst-stained total cells were calculated.

### Experimental design and statistical analyses

For the histological analysis of embryonic brains, at least three NF-YA nes-cko and control mice at similar embryonic stages were analyzed and observed reproducible degeneration phenotypes only in nes-cko mice. For comparison between two sample groups, data were first analyzed by F-test. For *p* < 0.05, the data were analyzed by unpaired Student’s *t* test (two-tailed); otherwise, data were analyzed by Welch’s *t* test (two-tailed). For multiple comparisons, the data were analyzed by one-way ANOVA followed by Tukey's post hoc tests using Prism software or R packages, anova, and TukeyHSD. No data points were excluded from the analysis. We considered the difference between comparisons to be significant when *p* < 0.05 for all the analyses.

## Data availability

All data are contained within the manuscript. Original ChIP-seq data have been deposited in GEO database under an accession number GSE244758.

## Supporting information

This article contains supporting information.

## Conflict of interest

The authors declare that they have no conflicts of interests with the contents of this article.
