# Supplementary material for: The transcription factor NF-YA is crucial for neural progenitor maintenance during brain development
Source: J Biol Chem. 2024 Jan 8;300(2):105629. doi: 10.1016/j.jbc.2024.105629 (PMC10839448; doi:10.1016/j.jbc.2024.105629)
Supplement: Supporting Figures S1–S8 [file mmc2.pdf]

Supporting information

The Transcription factor NF-YA is Crucial for Neural Progenitor Maintenance during Brain Development

Tomoyuki Yamanaka, Masaru Kurosawa, Aya Yoshida, Tomomi Shimogori, Akiko Hiyama, Sankar N. Maity, Nobutaka Hattori, Hideaki Matsui and Nobuyuki Nukina

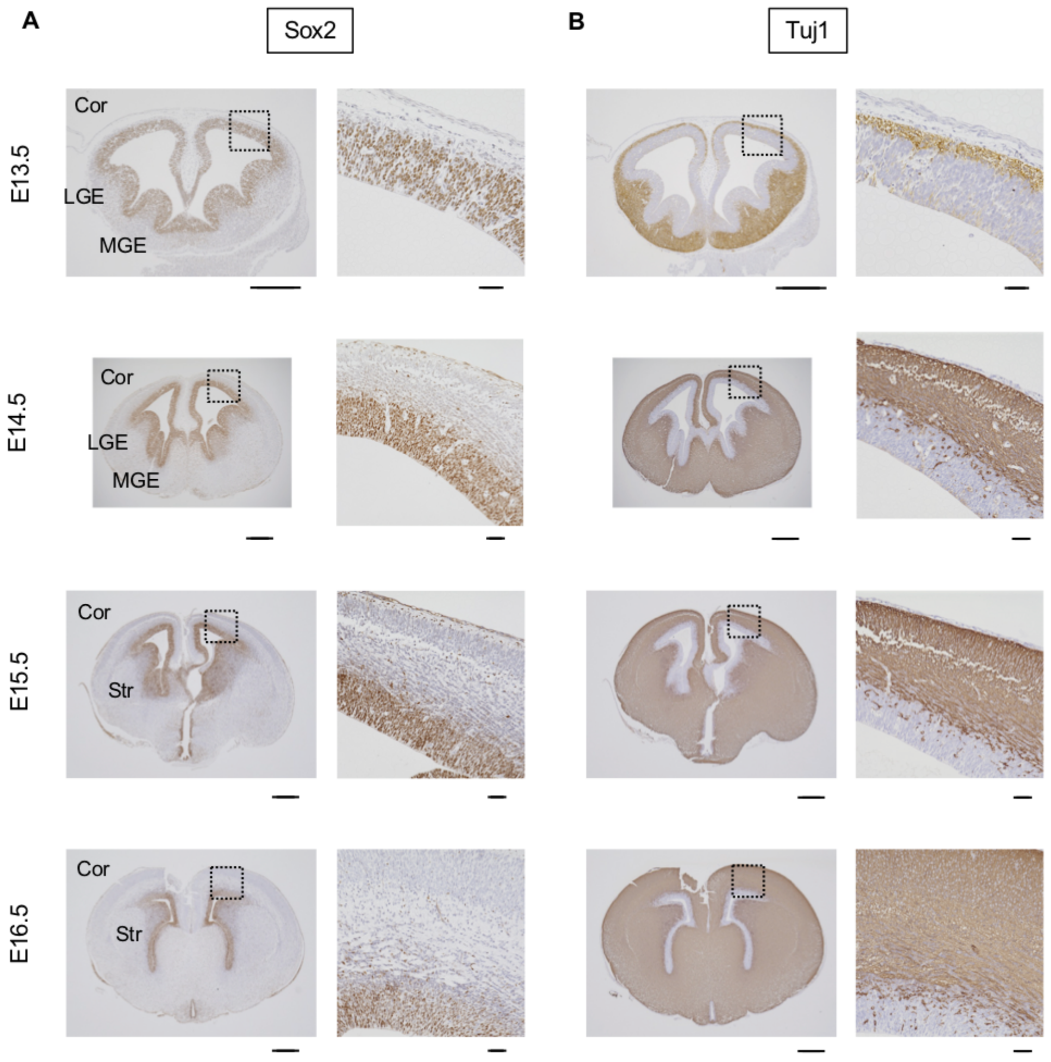

Figure S1. Immuno-staining of Sox2 and Tuj1 in developing brains.

Coronal sections from control mice at indicated ages were stained with antibodies (brown) for Sox2 (a pan-neural apical progenitor marker) (A) and Tuj1 (a neuronal marker) (B), followed by counter-staining with hematoxylin (blue). Cor (cortex), Str (striatum), LGE (lateral ganglionic eminence), MGE (medial ganglionic eminence). Magnified images of boxed regions are displayed in adjacent panels. The images of Sox2 (E13.5, E15.5 and E16.5) and Tuj1 (E16.5) are re-uses of the images in figures 2B, 1D and 1G, respectively. The scale bars are 500 µm (whole brain images) and 50 µm (magnified images).

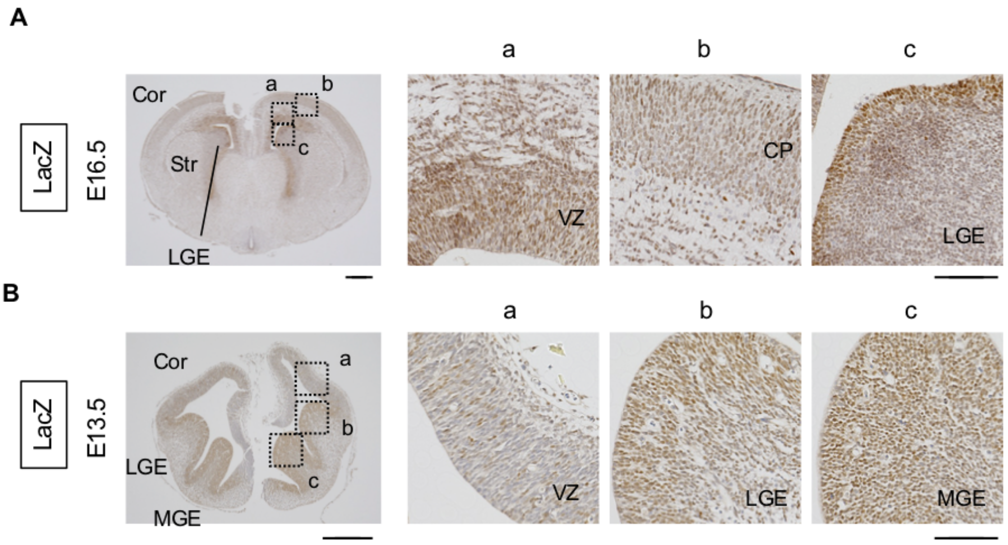

Figure S2. Immuno-staining of LacZ in RNZ reporter mice harboring nestin-cre transgene. Coronal sections prepared from control mice harboring RNZ reporter mice (NF-YA flox/+; nes-cre; RNZ) at E16.5 (A) or E13.5 (B) were stained with an antibody for LacZ. LacZ signals were highly detected in neural progenitors in cortical VZ and LGE at E16.5 whereas they were high in MGE, moderate in LGE but quite low in cortical VZ at E13.5. Cor (cortex), Str (striatum), VZ (ventricular zone), CP (cortical plate), LGE (lateral ganglionic eminence), MGE (medial ganglionic eminence). The scale bars are 500 μm (whole brain images) and 100 μm (magnified images).

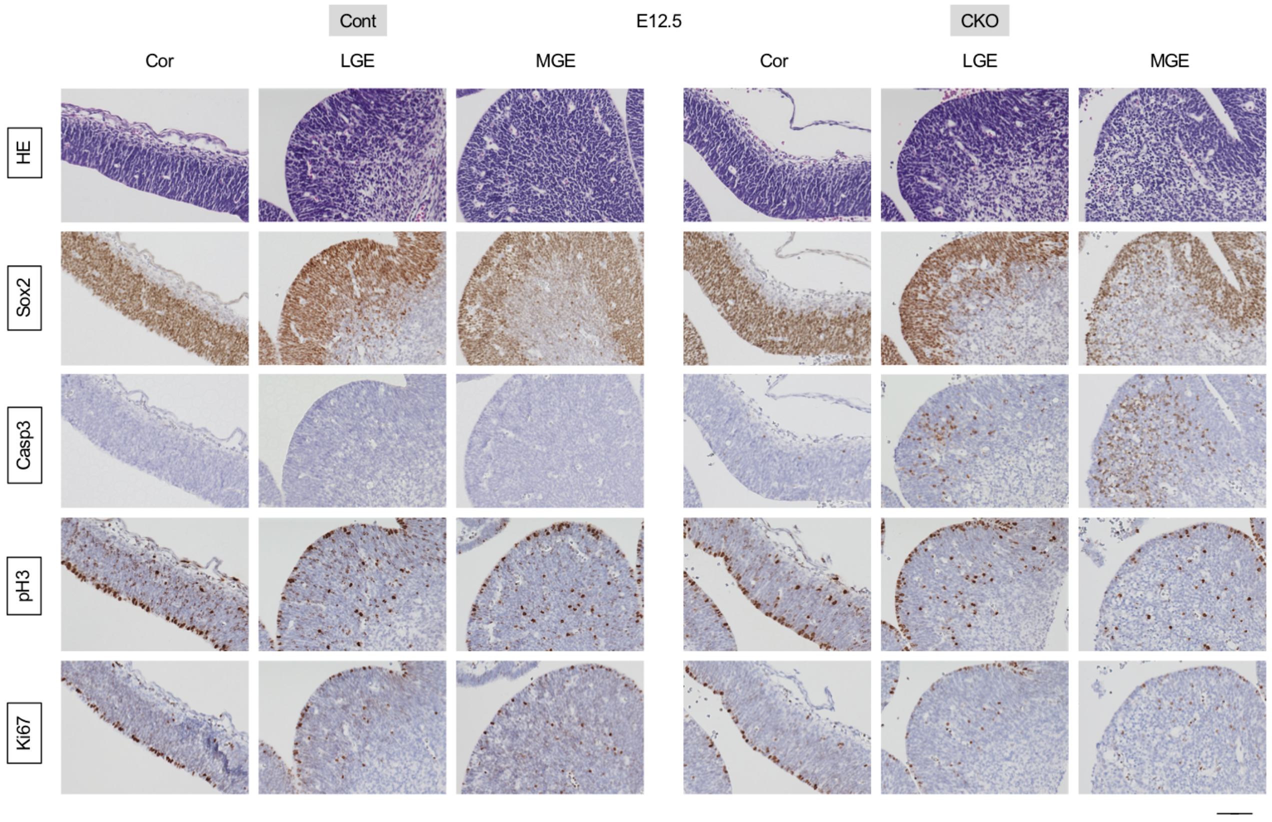

Figure S3. Immuno-staining of neural progenitors in E12.5 NF-YA nes-cko mouse brains. Coronal sections from NF-YA nes-cko and control mice at E12.5 were stained with HE, or immuno-stained with antibodies (brown) for Sox2, cleaved caspase-3 (Casp3), phospho-Histone H3 (pH3) and Ki67, followed by counter-staining with hematoxylin (blue). Note the loss of proliferating progenitors predominantly in MGE and relatively in LGE but not in cortex at this stage. This correlates with the presence of apoptotic cells. Cor (cortex), LGE (lateral ganglionic eminence), MGE (medial ganglionic eminence). The scale bar is 100 μm.

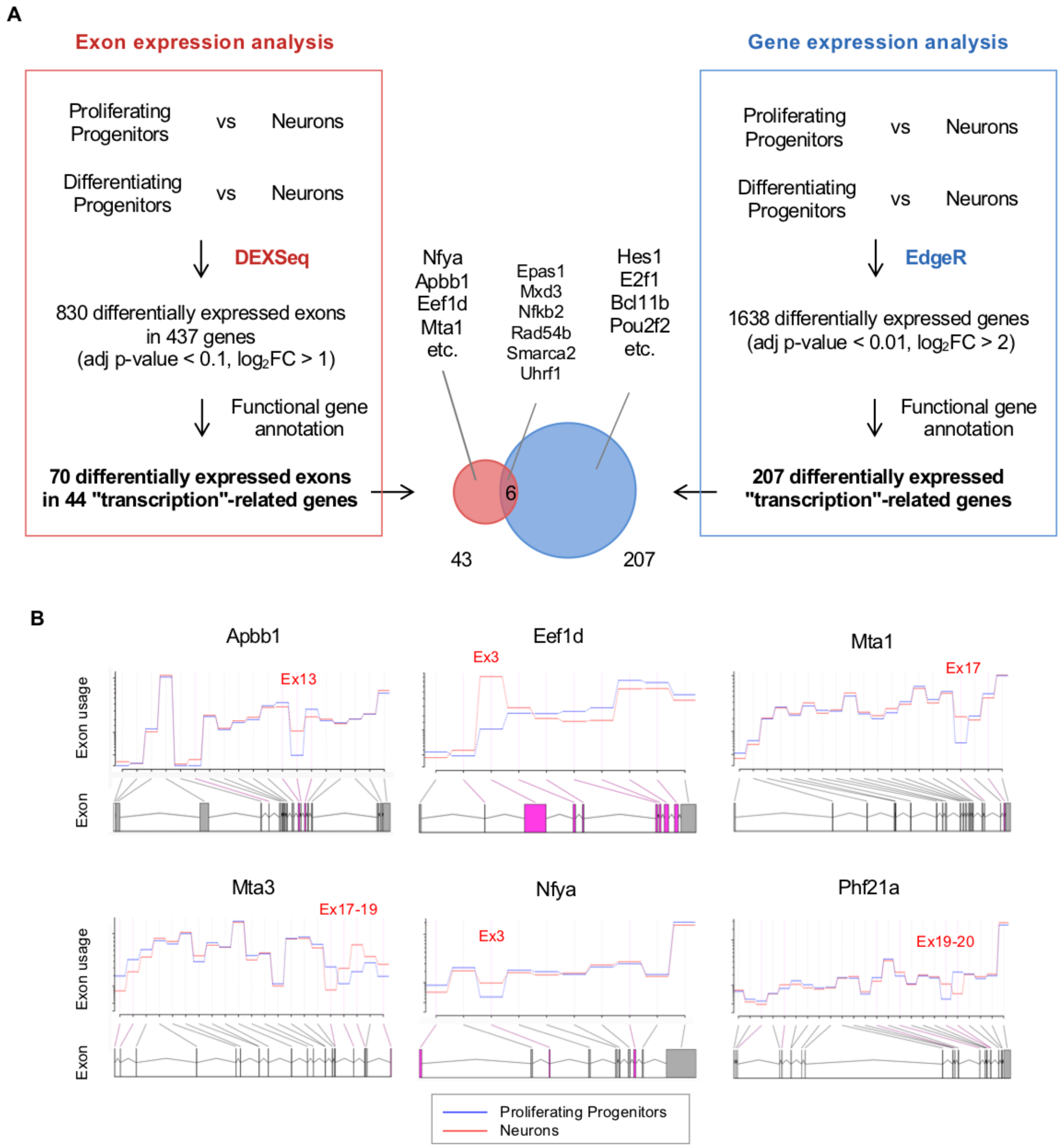

Figure S4. Screening of transcription factors showing altered splicing and/or expression during neuronal differentiation.

(A) Analysis by DEXSeq identified 830 differentially expressed exons in 437 genes, among which 70 were found in 44 transcription-related genes. In contrast, analysis by EdgeR identified 1638 differentially expressed genes, among which 207 were transcription-related genes. These were less overlapped and NF-YA was detected only by DEXSeq. (B) Example DEXSeq data of the transcription-related genes showing altered exon expression without altering gene expression.

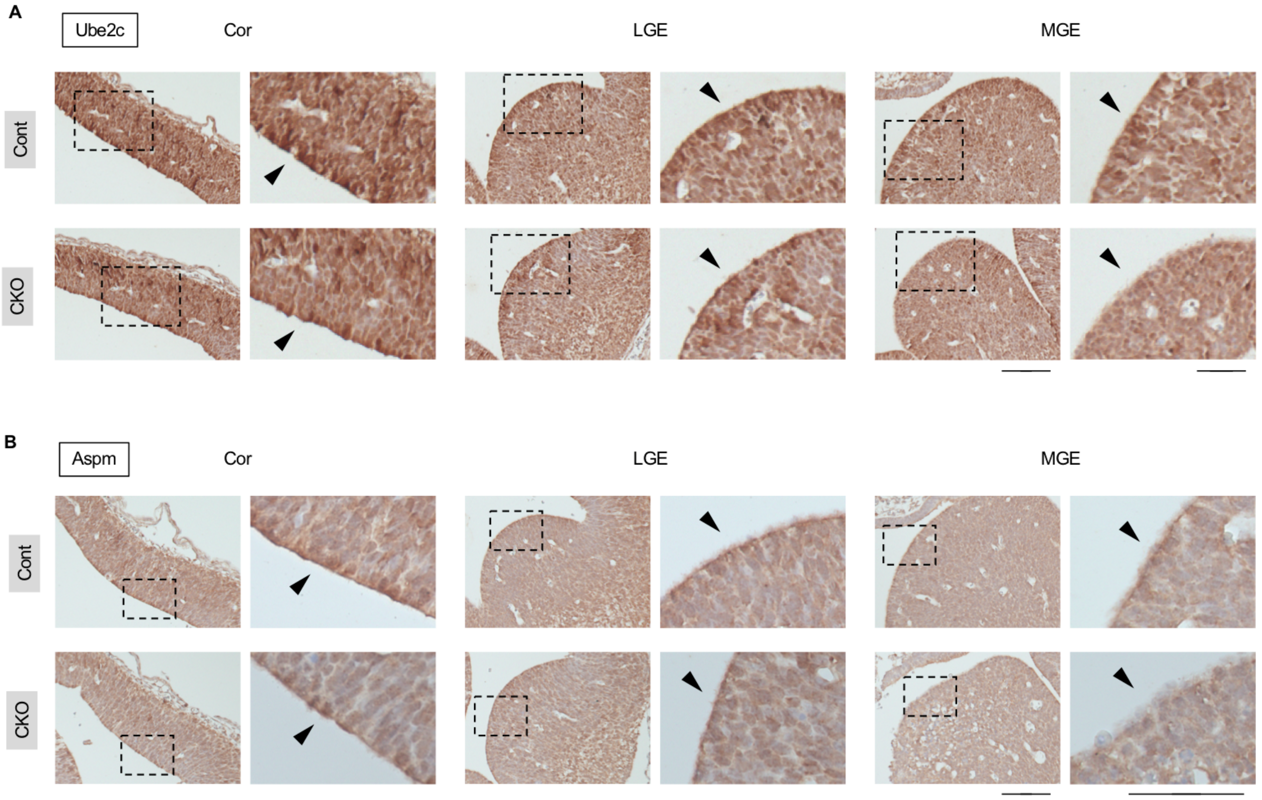

Figure S5. Immuno-staining of potential NF-Y targets in brain of E12.5 NF-YA nes-cko mice. Coronal sections from NF-YA nes-cko and control mice at E12.5 were immuno-stained with antibodies (brown) for Ube2c (A) and ASPM (B), followed by counter-staining with hematoxylin (blue). Magnified images of boxed regions are displayed in adjacent panels. Cor (cortex), LGE (lateral ganglionic eminence), MGE (medial ganglionic eminence). The scale bars are 100 μm (whole tissue images) and 50 μm (magnified images).

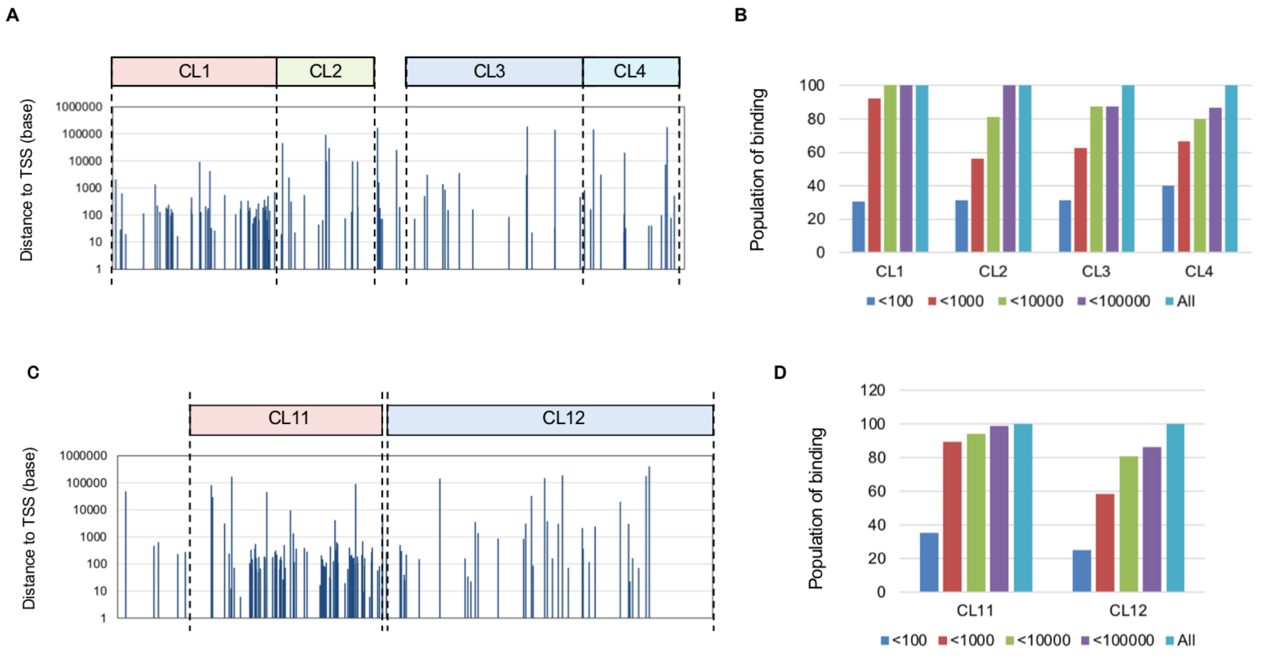

Figure S6. Gene annotation of NF-Y-ChIP peaks in distal -50 kb regions from TSS.

(A,C) NF-Y-ChIP peaks within the 50 kb upstream of TSS were gene-annotated, and peaks for deferentially expressed genes during neurogenesis (A) and corticogenesis (C) clustered in Figure 4 were picked up. Their locations from TSS are plotted. (B,D) Population analysis of the distances of the peaks in each cluster.

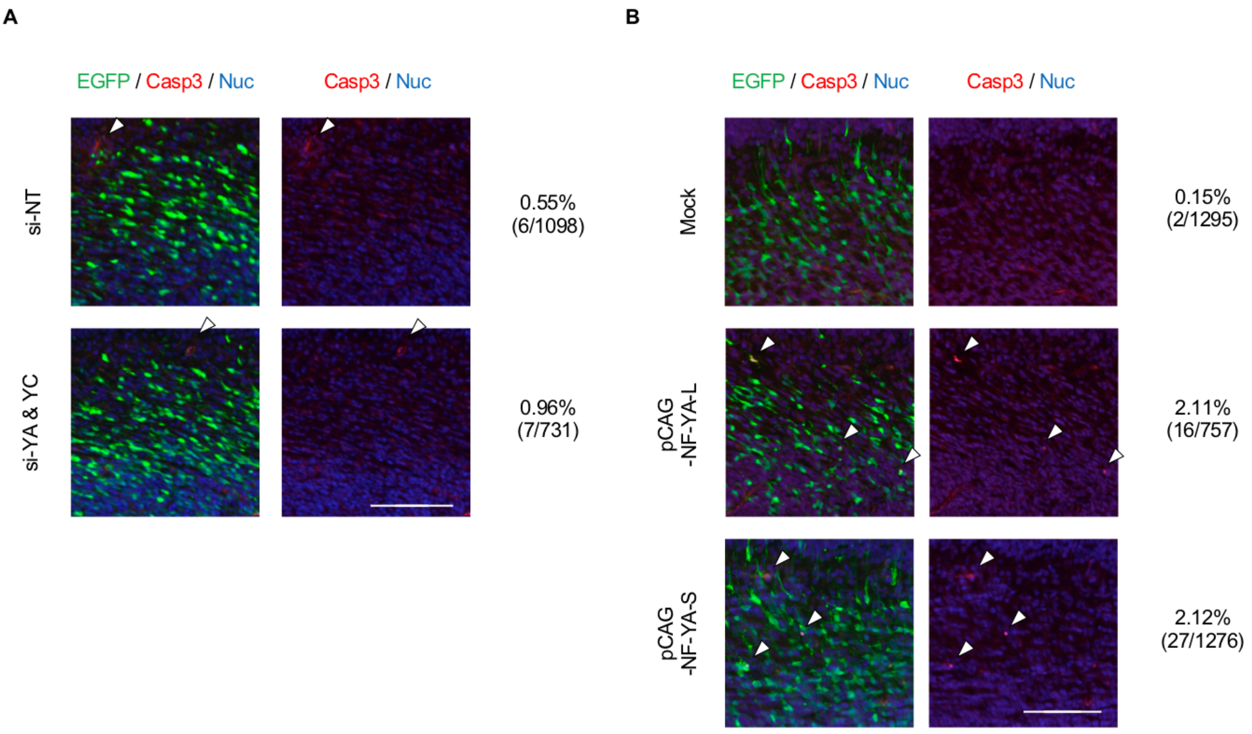

Figure S7. Cleaved caspase-3-staining of the brains subjected to *in utero* electroporation. Mouse embryos at E13.5 were subjected to *in utero* electroporation with siRNAs (A) or pCAG expression vectors (B). The brains were fixed after 2 days, followed by stained with cleaved caspase-3 (Casp3). Populations of the Casp3-positive cells among EGFP-positive cells are indicated. The scale bars are 100 μm.

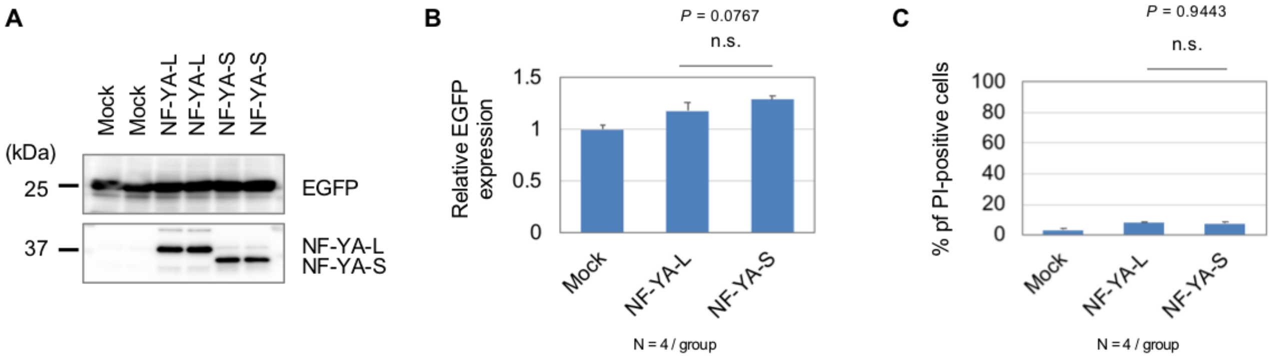

Figure S8. Western blotting and cell death analysis of transfected neuro2a cells.

Nuero2a cells were transfected with pCAG-EGFP together with pCAG-neo, pCAG-NF-YA-L or pCAG-NF-YA-S and cultured for 48 hr. (A) The transfected cells were subjected to Western blotting using antibodies against GFP and NF-YA. (B) Quantification of EGFP band intensities. (C) The transfected cells were stained with PI and Hoechst and populations of PI-positive dead cells among the Hoechst-positive total cells were examined. Values are means + s.d. of four data and statistically analyzed by one-way ANOVA followed by Tukey's post-hoc test (n.s.; not significant).
